# Life Challenges and Barriers to Help Seeking: Adolescents’ and Young Adults’ Voices of Mental Health

**DOI:** 10.3390/ijerph182413101

**Published:** 2021-12-12

**Authors:** Lisa Hellström, Linda Beckman

**Affiliations:** 1 Department of School Development and Leadership, Malmö University, 211 19 Malmö, Sweden; 2 Department of Health Sciences, Public Health Science, Karlstad University, 651 88 Karlstad, Sweden; linda.beckman@kau.se

**Keywords:** adolescents, life challenges, life skills, mental health, qualitative interviews, voices, young adults

## Abstract

Listening to the voices of adolescents and young adults regarding their lived experiences could be a way to identify important skills and abilities for adaptive and positive behaviour that will enable youth to deal effectively with the demands and challenges of everyday life. Hence, the aim with the current study is to explore the experiences and understandings of the life situation among adolescents and young adults of today, by making their voices heard in regards to mental health and help-seeking behaviour. A total of 6 group interviews were conducted with 22 adolescents and young adults (13 girls and 9 boys) ages 17–25 (M = 18.6 years). Data analysis was conducted using qualitative content analysis and resulted in two categories and five subcategories. The first category, Life challenges, included views on the sources of mental health, how to manage different types of relationships, and thoughts on accepted ways to express mental health problems. The second category, The need of present adults, highlighted important aspects for seeking help, such as an expressed need to be seen and heard by adults including parents, school staff, and other professionals as well as a need for adults’ increased availability. The challenges to students’ well-being and mental health are many, and there are no simple solutions. Based on the results in this study, life skills training should include elements to enhance the development of individual coping strategies, to be applied when life feels tough and when the body is experiencing stress reactions. Further, to minimize the risk of self-stigma and the internalization of negative stereotypes and self-blame, life skills training should include elements to increase knowledge of structural factors that have effects on the life situation as well as parents, school personnel, and other important adults.

## 1. Introduction

The number of young people experiencing mental health problems has increased dramatically. Approximately half of all 15-year olds in Sweden report recurring psychosomatic health problems, which is higher than any other Nordic country [1,2,3]. At the time of this study, a global pandemic of the coronavirus (COVID-19) has affected the health of people all around the world in different ways. Countries have adopted different strategies and restrictions to stop the spread, in many cases leading to isolation among adolescents and young adults, which, in turn, might worsen the already upward trend of mental illness. The long-term effects of the pandemic on mental health are under research [4]. The idea of a mental health crisis among young people is now firmly rooted in society and has been accepted by the public, politicians, officials, and practitioners who deal with young people’s health [5]. However, the state of mental health among young people is not clear-cut. While self-reported mental ill-health is increasing both nationally and internationally, levels of well-being are stable and serious psychiatric diagnoses have not increased significantly [6,7]. Recent research shows that 15-year-olds’ understanding of mental health (e.g., what it is, how it is manifested and where the distinction between mental health and everyday challenges should be drawn) is complex and variant [5]. Many young people also report a lack of willingness and knowledge of where to seek help if needed. Children and young people’s own voices are crucial to understanding their world and the aspects that contribute to their everyday functioning [8]. Children and young people are experts in their own lives and their voices need to be heard regarding their lived experiences in order to fully grasp the richness of their well-being experiences [9] as well as demands and everyday challenges.

Adolescence is a life period that involves many challenges and changes in different areas such as increasing academic demands, rearrangement of relationships with parents and peers, and developing one’s own personal identity [10,11]. According to PISA 2015 results on students’ well-being, 66% of students across OECD countries report that they worry about poor grades and 55% of students say they are very anxious for a test even if they are well prepared. In all countries, girls reported greater schoolwork-related anxiety than boys [12]. Another major concern of adolescents is relationships with peers [13,14]. According to Juvonen and Knifsend [15], teenagers look for strong social ties and value acceptance, care and support from others, and adolescents who feel acceptance by peers and that they are part of a school community are more likely to perform better academically and be more motivated in school. On the other hand, rejection by peers is more likely to lead to disengagement and decreasing academic achievement. Social environments are important contexts that influence how individuals behave and what they feel and think; as children grow older, the acceptance by peers becomes more important and their social relationships with parents and families are no longer perceived as so important [16]. An important social environment among young people is social media, i.e., internet applications that enable users to generate and exchange content with others (e.g., Facebook) [17]. Social media may serve as a key context through which emerging adults negotiate important developmental tasks, including identity development and maintaining social connections [18]. Previous studies among young adults (18–22 years) report associations between overall time spent on social media and ill-being [19,20] as well as the number of social media sites used and depression and anxiety symptoms [21]. Further, social media may function as a source of stress or reinforce negative self-evaluations through social comparisons, and heavier Facebook users are more likely to believe others are happier and have better lives [22,23]. Other studies highlight a multitude of positive experiences related to adolescents’ use of networked technologies. A recent large-scale survey among English 15-year-olds showed that moderate screen time is not harmful in itself but may actually be favorable in today’s connected world [24]. Despite a growing number of studies investigating the role of social media in the lives of young people, it remains unclear how various positive and negative social media experiences fit together [25].

A person’s ability to deal effectively with the demands and challenges of everyday life is manifested as psychosocial competence [26]. Psychosocial skills allow individuals to recognize, interact, influence, and relate to others and are related to positive mental health and well-being [26,27]. Life skills have been identified as abilities for adaptive and positive behaviour as well as an essential resource for developing psychosocial, emotional, cognitive, behavioural, and resilience skills to negotiate everyday challenges [26,28,29]. Hence, the nature and definition of life skills indicate that they are likely to differ across cultures and settings. However, a core set of life skills has been suggested through health promotion research on children and adolescents. These life skills are: decision making, problem-solving, creative thinking, critical thinking, effective communication, interpersonal relationship skills, self-awareness, empathy, and coping with emotions and stress [30]. There is a growing demand to educate adolescents with life skills to help them deal with their everyday challenges and transition into adulthood with informed healthy choices, but there are yet few qualitative studies on this subject [31]. The concept of mental health literacy refers to “knowledge and beliefs about mental disorders which aid their recognition, management or prevention” [32] (p. 182). Beyond knowledge and beliefs about mental disorders, mental health literacy includes “knowing how to seek mental health information; knowledge of risk factors and causes, of self-treatments, and of professional help available; and attitudes that promote recognition and help-seeking” [32] (p. 182). Many young people report a lack of willingness and knowledge of where to seek help if needed. Barriers to help-seeking have been identified to include a desire to handle the problem of one’s own, low mental health literacy, negative and stigmatising attitudes towards mental illness and towards help-seeking, amongst others [33,34].

School efforts aimed at teaching students (aged 4–18 years) about mental health and how they can manage their own and others’ ill-being (i.e., psycho-education) can reduce inward-looking mental problems [35]. However, investigating psycho-educational programmes in schools, Swedish research [36,37,38,39] has found a great discrepancy between what are considered the needs of young people from the viewpoint of the programmes and from the viewpoint of young people themselves. The focus of most programmes is often on young people’s individual thoughts and emotions, while young people highlight mental health as a complex social and relational matter [39]. 

### 1.1. Previous Research on the Perception of Youth Mental Health and Help-Seeking Behaviour

Definitions of mental health from the perspectives of the general public tend to focus on adult interpretations and show a discrepancy to the broader conceptualisation made by mental health professionals [40]. Similarly, discrepancies between adult and adolescent perceptions have been documented [41,42]. When asking children, they often show a great interest in mental health and can articulate what they want and need when it comes to support for mental health issues [43]. Factors influencing young people’s help-seeking behaviour include confidentiality and trust within the context of seeking help, perceptions of young people’s problems as generally less important than those of adults, and a heavy emphasis on internalizing or “bottling up” feelings as a popular coping strategy [44]. A recent Australian study found that there are discrepancies between adolescent descriptions of mental health on a conceptual basis and their representations of mental health relevant to their own lives and experiences [45]. This highlights the need to explore everyday challenges as experienced by adolescents and young adults in light of the increasingly prevalent rates of self-reported mental health problems. A Greek study found that pupils (13–16 years) described mental illness in a multi-dimensional way including, for example, being in a certain state of something; doing or behaving in a certain way; having psychological problems; not knowing what is going on around them; seeing everything in black and imagining things; and not being able to do certain things [46]. Considering that concepts of mental health are complex even for adults [47], a focus on students learning to understand and appreciate seemingly opposing ideas when considering mental health and illness may be a useful first step in the endeavour to develop mental health knowledge and understanding in adolescents and, in turn, in adults [45]. 

When it comes to research investigating youth’s perceptions on mental health, few are conducted on non-clinical samples [45]. The considerable uncertainty regarding the state of mental health among young people in general, their own understanding, and what are successful efforts to promote mental health, emphasizes the importance of including young people without diagnosed mental illness and their perspectives in mental health research [48]. Further, little is known what life skills can be identified as important to be able to handle the demands and challenges of everyday life. Despite a growing demand to educate adolescents with life skills to help them deal with their everyday challenges and transition into adulthood with informed healthy choices, there are yet few qualitative studies on this subject [31]. Discrepancies that exist between the views of adolescents and professionals are vital to acknowledge, considering the potential influence of such differences on the effectiveness of mental health policies and programmes that are developed by professionals but aimed at youth populations [45]. As young people’s actions are grounded in how they understand and interpret the world, understanding the perceptions and experiences about mental health and help-seeking behaviour is important in the undertaking of addressing mental health problems among adolescents and young adults [44]. In the current study, we listen to the voices of adolescents and young adults regarding their lived experiences and mental health. This will be an addition to previous research on young people’s perceptions of mental health, as we hope to be able to identify what skills adolescents and young adults need to be able to deal effectively with the demands and challenges of everyday life. Hence, the aim with the current study is to explore the experiences and understandings of the life situation among adolescents and young adults of today, by making their voices heard in regards to mental health and help-seeking behaviour. 

### 1.2. Research Questions

1. What skills among adolescents and young adults can be identified as important to be able to deal effectively with the demands and challenges of everyday life?
2. What are the needs and barriers to help-seeking among adolescents and young adults regarding mental health problems?

## 2. Materials and Methods

### 2.1. Data and Participants

This study is part of a larger project called “Creating better life skills among youth” and is based on data collected during spring and autumn of 2020. A previous research report with preliminary results from the study has been published [49]. Group interviews were chosen to obtain in-depth information from the participants; to encourage them to talk to one another, asking questions, exchanging experiences, and commentating on each other’s points of view; and to be able to ask follow-up questions when needed [50]. Besides group interviews with adolescents and young adults, group interviews with practitioners have been conducted within the larger project. This study involves participants in the ages 17–25 (M = 18.6 years) from four different cities in Sweden. Twenty-two adolescents and young adults (thirteen girls and nine boys) took part in group interviews. A total of six group interviews were conducted with three groups mixing boys and girls (*n* = 5, *n* = 4, *n* = 3), two groups including only girls (*n* = 4, *n* = 3), and one group including only boys (*n* = 3). Two group interviews were conducted face-to face, while the remaining four group interviews were conducted via the digital tool Zoom, due to restrictions connected with the global COVID-19 pandemic.

### 2.2. Procedure and Interview Guide

The study was approved by the Swedish Ethical Review Authority (No: 2020-01600). The participants were recruited via the Swedish national insurance company (Länsförsäkringar, LF, Stockholm, Sweden) in four different Swedish cities. LF consists of 23 regional insurance companies, located all over Sweden, that are committed to community involvement and work with social sustainability. Regional companies that showed interest in the study were asked to recruit young adults in the ages 16–25 to participate in the study. No particular selection criteria were stated. All adolescents and young adults who wanted to participate in a group interview were invited to the study. The participants were given written information about the study in advance and were informed that participation was voluntary, that their answers were anonymous, and that they could terminate their participation at any point. Written and oral consent were collected at the same time as the interviews. Since the participants were over the age of 15, no parental consent was needed. The group interviews were conducted using semi-structured interviews, and an interview guide was designed based on knowledge gaps identified in the literature review, namely making adolescents’ and young adults’ own voices heard regarding life challenges and barriers to help seeking. The main questions of interest were “What does health mean to you?”, “What skills do you need to handle life’s ups-and-downs?”, “Who do you talk to if you feel bad?”, “What do you think is missing to get more young to seek help if they feel bad?”, “What kind of help do young people need from adults to handle life’s ups-and-downs?”, “Where do young people turn when they need support concerning mental illness?”, and “There is a concept called life skills that is defined by the World Health Organization as abilities that make it possible to handle demands and challenges in everyday life in an effective way. What would you say are important life skills that a young person should have today?”. The semi-structured interviews were conducted with both authors, who took turns being the moderator in the sessions, present. The moderator asked the questions and followed up with questions such as “can you develop what you just said”, “what do you mean”, and “can you give any examples”. Before the group interviews ended, the participants were asked if they had anything to add or if they thought that something important had been left out of the conversation. The interviews lasted between 52 and 94 min. The transcripts were not shared with the participants prior to or following the analysis. The participants asked to take part of the results following publication.

### 2.3. Data Analysis

Data analysis was conducted using qualitative content analysis [51]. Each group interview was transcribed verbatim and quotations of relevance for the aim of the study were sorted to find patterns in the statements of the participants. Quotations in the results are labeled based on gender and municipality. When conducting qualitative analysis, it is paramount that the researcher maintains a vigilance and non-bias during analysis. This includes being transparent to enhance trustworthiness in data analysis, as well as being aware of one’s pre-understandings. Staying aware of one’s pre-understandings and expectations and putting these in a holding pattern while approaching data with an openness may lead to finding new perspectives [52]. Transparency includes being transparent about the rationale for the thematic structure in the coding process. In this study, transparency and non-bias were enhanced by including two researchers in the analysis process (the two authors) and by providing detailed descriptions as well as an example of the coding process (Table 1). First, the transcription of each group interview was read through numerous times by both authors and meaning-carrying units responding to the aim of the study were extracted. Descriptions of mental health and help-seeking behaviour constituted the unit of analysis. In the next step, the meaning-carrying units were condensed and abstracted into codes. In order to identify similarities and differences, the codes were compared and then sorted into subcategories. As the analysis proceeded, subcategories were subsequently clarified and adjusted and two main categories emerged (see Table 1). The initial coding of the transcripts was performed by the first author, and the coded data were examined by the second author for emergent subcategories. Comparisons were made with the context in each step of the analysis, to verify the empirical base of the data. The tentative codes and subcategories were discussed by both authors and revised until consensus was reached. What differed between the two researchers was their judgement about overlapping between content in more than one initial subcategory. In these cases, we returned to the meaning-carry unit to check if the meaning unit itself fit the subcategory or if the preliminary coding needed to be reconsidered [52]. A process of reflection and discussion resulted in agreement about how to sort the codes. The current study is reported in line with the Consolidated Criteria for Reporting Qualitative Research (COREQ) checklist [53].

## 3. Results

Analysis of the group interviews resulted in two categories and five subcategories (see Table 2). The first category (i.e., Life challenges) contains three sub-categories, namely sources of mental health problems, managing relationships, and an accepted way to express mental health problems. The second category (i.e., The need for present adults), contains two sub-categories, a need to be seen and heard and a need for increased availability.

### 3.1. Life Challenges

The category Life challenges emerged as important when listening to young adults’ voices about mental health. Within this category, three subcategories emerged: sources of mental health, managing relationships, and an accepted way to express mental health problems.

#### 3.1.1. Sources of Mental Health Problems

In the interviews with the young adults, several different reasons emerge as to why youth today experience mental health problems. The young adults reason about the increased figures for self-reported mental problems and discuss that one cause of concern may be young people’s changed living conditions and increased opportunities. As young people today are better off in general, they mean that the threshold for feeling bad has decreased. Another reason is described as all the choices young people today are faced with in school and which are perceived to affect their future.

*You’re too young to know… well “I want to be a carpenter, I want to be a doctor” and such. So you are left alone, sort of. You do not know where to go because you have to take certain steps to get to certain places. And then it’s kind of hard to decide, you do not know.*

(Municipality B, boy)

The young adults in the study express that they lack knowledge that is important for them to be able to make independent decisions. These may include choices that affect their future professional careers. This lack of knowledge that contributes to independence, they say, is a source of stress. They request information about life after school, such as what different professions entail, including the working conditions, and the advantages and disadvantages of different career choices. They believe that in the hunt for students, high schools do not always take the responsibility to market themselves in a truthful way.

*And I think maybe you should not paint a school with negativity but still present that if you work in these professions it will be like this… well it will not be that you work seven to five every single day and have weekends free and things like that… in healthcare. Now I also understood it when I applied for the program but no one told me about conditions you have during work, sometimes you do not even have time to eat lunch and everything like that. They paint everything that is so good and then this bad, you get it like a slap in the face, because no one tells you about it. Because it’s criticism like ... And then you get these bad things for free, you get to learn it yourself as well.*

(Municipality B, girl)

There was an expressed desire that the schools should contribute with an increased knowledge about life after school and all that it entails. The young adults experienced that as soon as they leave school they are expected to know how to acquire a home and take a loan from the bank, what insurance to have, how to pay bills, and how to set up a budget. They also expressed that independence presupposes good self-confidence, so that one is able to take care of oneself. It also emerged that to be able to become independent it is important to have a fair picture of what adult life means and that you do not always receive this support from home.

*I think there must be a changed picture of what life after school really is, when you are in school. To find out all that yourself afterwards, it becomes very difficult for very many depending on what kind of support you have or if you have parents who support you or something like that. Because already in high school, they say that "now all the responsibility lies with you" and then you turn 18 and then you are an adult even though you are not treated like an adult.*

(Municipality A, girl)

Constant comparisons with peers are another thing that creates stress and situations of mental problems according to the young adults. Comparisons are made on social media, where much concerns painting a flawless picture of yourself, partly to show how you have succeeded in different ways in life but also so that your friends will not become tired of you. The young adults say that it is easy to compare themselves with friends and acquaintances on social media and wish that they had the simple lives that are often illustrated there. It can be about looks, clothes, make-up, training, earning a lot of money, and establishing a career. If it feels that a young adult cannot achieve all this, they beat themselves down. The young adults also described that youth are influenced by various influential people on social media, influencers. Staying up to date with what the popular influencers’ post online is also expressed as a source of stress.

*“So there’s a lot you have to live up to, like… what they post, that this is what life should look like. If you do not have this instagram life then life is no good”.*

(Municipality B, girl)

Comparisons in school are also listed as a factor that, through stress and pressure, contributes to young people’s well-being and mental health problems. The comparison between schoolmates can lead to many young people feeling bad.

*A classic example is this… well after the test… that many people like to walk around and ask "what score did you get?" and then you feel pressure if you did not reach the same level or so. And that in turn can lead you to feeling unsuccessful and then you skip the next test instead.*

(Municipality C, girl)

It appears that young people who perform worse than their peers, as well as young people who perform better than their peers, experience stress and pressure similar to each other. According to young adults, youth today place high demands on themselves to perform in school, which can sometimes be unreasonable. There is also an experience that teachers do not always have the time to help when they feel bad about school. Some of the participants in the study also make the connection between mental health problems and neuropsychiatric diagnosis. They discuss that underlying diagnoses that remain undiagnosed can be a reason why some young people today feel bad, since they end up at a disadvantage because the expectations and requirements are not adapted to the difficulties that the diagnosis entails.

*That’s how it was for me and I got depressed from a very early age so there was no one… I did not understand and there was no one around me who saw it either and I think I pushed myself quite far... for me it took quite a long time to understand that it was a state of disability in a way that you could get well from it and that it was not something that was going to last forever.*

(Municipality A, girl)

The young adults describe situations where they have experienced failures and that this is something that feels very hard to handle. What is described as hard is to fail in front of others, especially peers who you do not know so well. Strategies to handle tough situations are discussed and include learning how to handle that failure is a part of life. This includes an increased understanding that it is okay to feel bad sometimes and an increased understanding that you will face adversity in life.

*I actually believe that... you kind of have to see the real world, the more you get into it… so you do not try to change or remove that which is experienced as difficult, because you encounter some hurdles.*

(Municipality D, boy)

An increased understanding that life is not always on top could, according to the young adults, also mean that you become more accepted regardless of how you feel and the reasons behind it. They believe that an increased understanding could also mean an increased insight into the fact that there is help available to help you handle your mental health problems.

#### 3.1.2. Managing Relationships

School is the young people’s arena, where most of their everyday lives take place and it emerges that school-related stress includes both demands to perform well in school and to create and maintain good peer relationships and love relationships. A source of stress, according to the young adults in the study, is young people’s own thoughts about what are normal thoughts and feelings in relationships with their partner, peers, or parents. One boy puts it:

*So I think love relationships are after all… when it becomes relevant, it takes up a very, very large part of one’s thoughts and person in general, I think. And to just be able to go to someone and ask “am I doing the right thing? Am I reasonable? ” such things, would probably have been very… a greater security I think than to deal with all this myself.*

(Municipality A, boy)

When it comes to relationships, it arises that something that affects one’s own state of mind is the mental state of one’s friend. Tools to handle their friends’ mental health problems and where to turn to obtain help are requested by the youth.

*My mental state right now is because everyone comes to me to talk and I’m the one who likes to be quiet and not talk so much as well. So it would be nice to have tools to like....*

(Municipality C, girl)

According to the young adults, there are clear norms dictating how boys and girls should behave. These norms advocate a "macho culture" among boys where boys should not cry or open up too much about their emotions. Girls, on the other hand, are allowed to be more sensitive. It was perceived that it is tough for boys, as they had to behave in a certain way in school to fit this norm.

*You are expected to be masculine, to be rock hard, you should endure everything like this. If you say something like “I’m not feeling well today, it’s not going to work today. My psyche destroys everything ”. It is not an option for them, then it is like “pack your bags and don’t come back no more”. So you must never lower your guard or what to say and calm it down. It is the one who is most masculine who dictates the rules.*

(Municipality B, boy)

Another norm that boys and girls talked about is that boys should not perform academically well in school. Boys should perform in other ways and that in some contexts it is not accepted, or perceived as nerdy, to keep studying after high school. This could lead to a vicious circle where boys feel bad about bad grades. However, there was a perception that the macho norm was not as strong now as before but that it was still prevalent. The perception was also that boys and girls experience mental health problems equally and that relationships and school were something that both boys and girls experience as important areas and that affected their well-being. For girls, it was perceived to be more acceptable to talk about situations involving mental health problems, while boys were perceived as good at hiding their mental health problems.

*All my guy friends from primary school have told me that they experience situations of mental health problems. And it’s a bit like "yes but why don’t you talk about it" and "no we never talk about that". So guys do not talk about feelings like we girls do.*

(Municipality D, girl)

#### 3.1.3. An Accepted Way to Express Mental Health Problems

Norms and expectations on how to talk about mental health problems were expressed in various ways. On the one hand, there is a picture that there is still a lot of stigma about mental illness. Young people only talk to their closest friends about how they feel and it is still not perceived as acceptable to feel bad. The young adults in our study talked about how people with mental illness can be perceived as lazy, that they are labeled as attention seekers, or that they are perceived as strange. The perceptions are that there are stereotypical images of how certain diagnoses are expressed that do not always give a correct picture, which means that it can be difficult to be taken seriously. A participant who had been diagnosed with Attention Difficulty Hyperactivity Disorder (ADHD) says:

*Students could sit in the classroom and say “but I can not focus because I have ADHD” and scream and have fun, which made it harder for me who actually had the diagnosis… because I know the people who sat and screamed did not have it… it was a way to blame someone else or scream or get yes… it was like, it was not true, which made it very difficult for me and the other people in the class to be taken seriously. And the same with anxiety and depression, it unfortunately becomes difficult to be taken seriously because it is based on these stereotypes that exist around it.*

(Municipality A, girl)

It emerged that there is an understanding among youth that many young people today have mental health problems. However, at the same time, the young people question whether this is really the case and mean that it is hard to determine who really suffers from mental health problems. It seems like it has become normal, a norm, and that many young people are not afraid to express this:

*“But now... it’s a little more mainstream to have mental health problems”.*

(Municipality A, girl)

The young people believe that, after all, it has become more accepted to say that they suffer from mental ill-health. In some cases, they describe that it may be that many young people voice their problems as a way to gain attention from those around them. One boy problematizes this:

*It depends on the way you seek attention. If I go out and shout that I feel really bad, then it’s like a cry for help but then I do not do it the right way I do not think”.*

(Municipality D, boy)

It emerges that perhaps it is not feeling bad that is accepted or not, but what you feel bad about. The response that young people think they will receive from those around them affects whether they choose to tell someone how they feel. It emerges from the conversations that many young people choose not to tell anyone if they experience mental health problems. The reason is, among other things, a fear of not being accepted or of being taken seriously if you open up about how you feel. If you normally are perceived as a happy and positive person, you do not want to risk this image change. At the same time, they reflect positively if someone close to them would tell them that they are suffering from mental health problems. Another reason to not tell anyone is that they do not want them or their problems to be a burden for anyone else or that they do not want to risk being treated with diminution:

*“I think the difference is a bit, for me anyway, that what you often hear is "come on, pull yourself up" and they can do it… there is a difference as well. It’s not as simple as that”.*

(Municipality A, girl)

### 3.2. The Need for Present Adults

The young people in the study expressed a need for present adults around them, such as parents, school staff, and other professionals, as important for them to want to seek help with issues relating to mental health. The category The need for present adults was expressed as a need to be seen and heard and a need for increased availability.

#### 3.2.1. A Need to Be Seen and Heard

To increase young people’s help-seeking behaviours, there was a general demand for a greater understanding of what mental illness is and a request for greater respect towards those who experience situations of mental health problems. One girl says that she would rather have her feelings confirmed instead of hearing that everyone is having a hard time at times:

*Of course you understand that not everything is a bed of roses, but sometimes you have to be confirmed that if you do not seize it now, it can get worse. So you can go from having just a bad day, then there will be several bad days and you only hear that everyone has bad days sometimes. So sometimes you need confirmation for… and understanding as well.*

(Municipality B, girl)

It emerged that the young adults expressed a need for parents to be more interested and ask them how their day at school had been. When parents asked the same questions every day, they often received the same answer. A girl says:

*I usually feel that way when parents ask the same question every day, then I feel that it becomes monotonous because then it’s the same answer every day as well. So there will be no progress. So they will not find out so much more than they already know. Yes, but "how was school today?" It was fine ”. Yes, but they do not ask any more questions "well what did you do?" "The ordinary".*

(Municipality A, girl)

It was also expressed that adults should not be afraid of being wrong, or of wanting to learn from young people, for example, when it comes to social media, to show interest and to not be judgmental. A girl says:

*We are supposed to learn from adults, that they have more experience in life and they know what is right and wrong, but then, they must also learn from us. It is we who live in the youth now.*

(Municipality D, girl)

The young people sought more acceptance from adults and to not be questioned about their actions. For example, when it came to actions on social media. They thought it was based on parental ignorance and that they themselves had not been a part of social media when they were growing up. It was perceived that adults could easily become hysterical but that it is important that the adults trust their children and instead talk to them if they felt that something was wrong. Some felt that they had been wrongfully diminished when they told adults about feeling bad.

*I would also say diminishing… that people say "pull yourself up, bite the bullet” or “everyone has bad days and hard times" and then you sit there and feel stupid for feeling the way you do when it may not be quite the same as for those who do not feel mentally ill. But then you feel stupid or yes, you are kind of diminished and I do not think people think about it. But it reflects quite hard on oneself. And then maybe you start telling yourself that "I’m just lying, I’m totally weird in the head”. So it will be like a huge mistake, which is a great pity because maybe you should not have to hear that.*

(Municipality A, girl)

Of importance for the young adults in our study to seek help turned out to be a sense of trust in the different professions they encounter in different situations. The youth experiences were that the meeting with professionals is not always based on the young people’s needs. Among other things, there was a need for the conversations to be forward-looking, where they themselves could talk about their experiences, and not become too caught up in the causes behind the ill health that they themselves do not always perceive as relevant. A girl says:

*So I have a psychologist that I also go to at BUP [child and adolescent psychiatry] but I don’t really feel… so we have not really got that connection or what to say, that I feel I can talk to her. Because it feels a little, for me it feels very well… so… that there must always be a solution or a cause and you always have to go into all these causes and for me it just feels like… so, I would rather move forward if you say.*

(Municipality A, girl)

Trust in professionals is also about an experience of being met by an understanding that in the process of feeling better, one is also allowed to fail. A girl tells of a situation where there was a lack of trust in a counselor and she ended up lying in an attempt to achieve the expectations placed on her because she did not feel that it was acceptable for her to fail. Feeling seen was also something that affected their sense of trust. To be seen also meant to be treated in a respectful way by the professionals, reflected in how they communicate, both with the youth themselves and with other professionals who are familiar with the problem.

#### 3.2.2. Need for Accessibility

There were participants who felt well informed about where to turn for help, but the voices parted and there was also a desire for more information about where to turn when experiencing mental health problems. This also applied to the school counselor and school nurse, but if you wanted to meet someone outside the school, it was not as clear how to obtain help with questions that may not qualify for specialist help. There were suggestions, such as information about the youth clinic as an anonymous activity from which you can obtain support; or more information about what the Student Health Team could assist with, more specifically and continuously; or an information letter sent out to all students, or perhaps directly to their parents, about where to turn for help. Another aspect that was raised (here by boys) was that when informing about where to seek support and help if you feel bad, this should be specially designed to also reach out to boys. One boy expressed:

*“It feels like they have to come and make some contact with you… they may be able to ask like everyone else, of course it is difficult but then ask how they really feel …”.*

(Municipality A, boy)

One suggestion that came up was to offer individual dialogues with the school social worker each semester, like a form of screening. This would reduce the stigma of contacting the social worker yourself. At the same time, they understood that it could be an unreasonable amount of time to put on the school social worker. It was perceived as a big step to seek help when experiencing mental health problems. There was an experience of feeling lonely in what they were going through, something they were ashamed of, and that in today’s society it is still not perceived as normal to have mental health problems. A girl said:

*“It is still not normalized, it is just made up”.*

(Municipality C, girl)

The young adults discuss that a visit to the school social worker should not be visible to others, and it would be better if they could visit the social worker after school hours, more discreetly. There was a perception that those who went to the school counselor had real problems, which was perceived as shameful. Despite the big step, it was perceived as something positive to obtain help for your problems. It was also important to obtain help early, so that the situation would not develop too far and you kept things going for too long. It was expressed that they hoped that someone in their surroundings would see them before the problems would become too big. However, there were perceptions that if you have problems, you must be the one who seeks help because people in the surroundings are bad at reading the signs or are afraid of bringing it up because they do not know what the reaction could be. Even if a person who has problems knew about the possibilities of seeking help, they still had to make the contact themselves and this could be a hurdle:

*“That you yourself have to reach out a hand when it’s me who needs the help”.*

(Municipality B, girl)

One girl believed that adults at her school were good at talking about mental health problems. She gives examples of how the school nurse and the school social worker usually make sure to visit and have time for each class from time to time and ask "how are you?" and talk about how they work and how they can help the students.

Reasons why their need for support was not always met was explained by the young people in some cases as a lack of resources and stability. The young people realize that they cannot always obtain the help they need because the student health professionals do not have the time or resources to meet the needs of all young people. This leads to the idea that different matters must be prioritized over others. The young people thus express an understanding that their needs cannot always be met due to the fact that the resources simply do not exist. A girl says:

*I also remember that the school social worker who were available, they were maybe there once a week, every other month as well so there was no opportunity to go and talk to them. They sat there at school for three hours one day a week and I don’t really know how it would be prioritized who would be allowed to go and talk to them.*

(Municipality A, girl)

The need for stability was expressed as the importance of having adults around them with whom there was an established lasting relationship. In this way, it would not be up to the individual student to tell their story every time new staff start at school or in the student health team. The participants attach great importance to the school as the primary support and believe that the school should become better at intervening early. A boy says:

*I think this is very important for school social workers… at [the school] where I go, where it was changed… so we could have the same school social worker for two months tops. And then there are those who have problems and may want to go and talk to a counselor… then they never have the time to develop a trust in this person. So I think it is very important that you have this adult… that you can talk to and you can have the stability over time.*

(Municipality D, boy)

## 4. Discussion

### 4.1. Results Discussion

The aim with the current study was to explore the experiences and understandings of the life situation among adolescents and young adults of today by making their voices heard in regards to mental health and help-seeking behaviour. The specific research questions were (1) what skills among adolescents and young adults can be identified as important to be able to deal effectively with the demands and challenges of everyday life, and (2) what are the needs and barriers to help-seeking among adolescents and young adults regarding mental health problems. In addition to previous research, this study uses a nonclinical sample to explore broader perceptions of everyday life challenges among adolescents and young adults without an outspoken mental illness. The increasing number of young people reporting mental health problems and stress is a major concern that needs to be taken seriously [5]. The results of this study may guide actions taken to promote mental health and prevent mental ill-health on a broader level. The main findings showed that the participants in our study present two different sides when it comes to mental health problems. First, mental health problems are described as mainstream and something that everyone is experiencing at some point. Second, adolescents and young adults also experience stigmatizing attitudes and other barriers associated with mental health, which results in few seeking help when they really need it. In addition to previous research on stigma, this study indicates that focusing on reducing self-stigma (i.e., the internalization of negative stereotypes and beliefs about mental illness held by others) may be of equal importance when working to improve life skills among youth. By listening to the voices of adolescents and young adults regarding their lived experiences and mental health, this study will be an addition to previous research as we have strived to identify what skills adolescents and young adults need to be able to deal effectively with the demands and challenges of everyday life.

#### 4.1.1. Life Challenges as Identified by Young Adults

Different challenges in the lives of young people emerge in the discussions. A major life challenge was expressed as striving to always be on top and the consequences of failure were described as devastating, in the worst case leading to mental ill-health. To be able to meet these challenges and setbacks, young people may need to develop individual coping strategies and relevant life skills. By focusing on the development of important life skills early in life, young people may be better prepared and equipped to meet challenges in life to prevent developing mental ill-health. Life skills have been identified as abilities for adaptive and positive behaviour and as an essential resource for developing skills to negotiate everyday challenges [26,28,29]. In this study, the most pronounced life challenges voiced by the young adults included academic failures, negative self-evaluations through social comparisons, relationship problems, and other performance-oriented tasks that result in stress and negative feelings. This type of stress usually finds expression in bodily reactions such as increase in heart rate, sweating, dizziness, or nausea [12]. The expressed sources of stress are in line with previous research indicating that adolescence is a life period that involves many challenges and changes in different areas such as increasing academic demands, rearrangement of relationships with parents and peers, and developing one’s own personal identity [10,11]. An important coping strategy and life skill that emerged among the participants was more knowledge related to how their bodies react to stress, and that these are normal reactions. Further, it emerged that what is being portrayed on social media creates an image that life should be good every single minute—and that constant comparisons creates feelings of failure. This is in line with previous research showing that social comparisons on social media may lead to beliefs that others are happier and have better lives [23], which may promote anxiety symptoms and cause interference in daily functioning [19]. In addition, the young adults in our study express a need to receive information about how to handle life challenges without having to gain the knowledge through their own lived experiences. Many times, adults’ willingness in “trying to fix the problem” may disable youth to find their own coping strategies. Youth may need to be exposed to setbacks and experiences of failure, and not try to avoid them, in order to develop independent coping strategies. In accordance with Wickström and Kvist Lindholm [5], youth, as well as adults, need constant reminders that it is a part of life to feel sad, angry, or upset from time to time, and that these feelings are not harmful and do not necessarily indicate mental ill-health. Children and youth that are able to use a broad range of strategies in an appropriate way to face demanding situations are likely to show a more adaptive psychological functioning than young people who use fewer coping strategies [54,55]. 

Many of the life challenges identified by the young adults in the current study are related to the youth themselves. Studies show that young people’s description of mental health problems and difficulties in everyday often are related to school environment and workload, relationships and social norms, and bodily experiences and body-ideals [48]. There is a risk that the reporting of young people’s mental health in the media and elsewhere focuses on individual factors, which shifts the focus away from more structural explanations within the school system’s organization and problematic living conditions. Further, there is a risk that alarming messages in the media and elsewhere in part will negatively affect young people’s self-image and belief in the future negatively, which can lead to a focus on care and medicalization instead of prevention [56]. Young people living in difficult circumstances may see themselves as the problem and interpret feelings of irritation and depression and similar symptoms as feelings they should remove at any cost [56]. Increased knowledge of these structural explanations may be helpful tools for youth and adults to understand their whole situation so that they can find constructive coping strategies. Listening to the voices of young people, it is evident that there is a great need for youth as well as adults to gain more knowledge about mental health, its risk factors and causes, coping strategies, how to seek information, and where to seek help, that is, strengthen their mental health literacy [32]. 

#### 4.1.2. Barriers to Help-Seeking Behaviour

The young adults in the study expressed a need for adults to see and hear them, including a greater acceptance and respect for their situation and a sense of trust. To meet youth in a respectful way and to signal that they are seen and heard could be a way to confirm their feelings and show an understanding of their situation, to show interest, to not be judgmental but willing to learn from them. This indicates that adults (including parents) could benefit from increased knowledge relating to mental health literacy and access to communication tools that will increase their ability to meet the youth and talk to them in a constructive way [57]. Barriers to help-seeking have been identified to include, amongst others, a desire to handle the problem of one’s own, low mental health literacy as well as negative and stigmatizing attitudes towards mental illness and towards help-seeing [33]. The unwillingness to seek help among the youth in our study was expressed as a way to risk your image, while there was also an expressed gratitude and admiration towards close friends voicing their problems. Underlying expectations expressed as norms, attitudes, and values are visible in the interviews with the young adults in our study. All of these outspoken and unspoken expectations relating to youth behaviour affect the understanding and help-seeking actions taken among youth [58]. 

It is often seen as a giant leap to set your foot in the school social workers’ office, indicating that you need help and that you have problems. People with different forms of mental health problems often experience self-stigma, whereby they internalize negative stereotypes and beliefs about mental illness held by others leading to feelings of shame [59]. Hence, self-stigmatization may decrease youth willingness to seek help [60,61,62]. A literature review identified psychoeducation as the most common intervention strategy to reduce self-stigma related to mental illness [63]. While it emerged that some youth are good at verbalizing their problems on social media, it was also evident that expressing your problems too openly was seen as a way to seek attention and was looked down upon. Hence, there seems to be many barriers for youth to seek help for their problems that can be related to the personal image they want to portray [61,62] as well as the unavailability of resources [33]. Research shows that many children do not obtain the help they need in time [64], which could be a reason we see that more children turn to social media to express their problems for which they receive attention and concern. Further, social media may function as a maladaptive coping strategy, as individuals may use social media sites to avoid real-world stressors via their distracting features or posting about their problems [19]. 

The barriers to help-seeking need to be lowered, and there is an outspoken need for adults to be more present and available. The young adults in the current study expressed that the student health team was perceived as unavailable making it hard to seek their help. Different ways to lower these barriers could be to invest more in outreach activities, such as individual and recurring dialogues with the school social worker or forms of help-seeking that avoid disclosure. The young adults in this study saw these activities as ways to reduce the stigma and pressure of contacting the school social worker yourself, or to be able to keep your help-seeking unrevealed. Web-based platforms could be seen as a form of outreach activity where youth could avoid disclosure [65]. Self-help interventions are also accessible to anyone, including individuals who would otherwise avoid seeking help due to fears about disclosure [59]. For more youth to seek help, it is reasonable to think that they must believe that it is worth the investment in time, commitment, and emotions.

### 4.2. Strengths and Limitations

This study used a small sample of adolescents and young adults, so the results may not be directly generalizable to other countries or age groups. Despite employing a nonclinical sample, several participants expressed personal interactions with counsellors, doctors, and psychologists in relation to mental health concerns, which may have influenced their views. Further, the global COVID-19 pandemic, and its associated restrictions and isolation for this target group, may have had some effect on their views on mental health. The current study used group discussions to encourage active discussions. The group interaction offered by group discussion encourages people to talk to one another, asking questions, exchanging experiences, and commentating on each other’s points of view [50]. The aim was not to study a gender perspective, and the group discussions were conducted as same-gender discussions as well as some groups with mixed gender. Although there were a few more girls in the sample compared to boys, the perception among the participating researchers was that neither the varying group compositions nor the uneven gender distribution affected the discussions in the groups or the results in any ways. The age of the participating youth (17–25 years of age) may have made them less susceptible to being affected by discussing a sensitive topic such as mental health with the opposite sex [66]. However, mixing ages in the different groups made it impossible to draw any conclusions regarding differing views from a developmental perspective, which could be seen as a limitation. Choosing girls and boys from different cities and in different ages may have enhanced the credibility of the data, as it offered a richer variation and understanding of mental health among youth with different backgrounds. Another limitation might be that we used Zoom to perform some of the interviews. Natural pauses might become rare and forced. We would have preferred face-to-face interviews, but this was the only option during the restrictions at the time. On the other hand, using telephone or other techniques such as Zoom might increase feelings of anonymity, making respondents more relaxed and open, which in turn can decrease interviewer effects [67]. Some of the participants chose not to show their picture, which made them more comfortable in sharing. Further, the trustworthiness was enhanced by involving two researchers in the analysis process to reach consensus and by including quotations from the transcribed text showing similarities within and differences between categories [51]. To use young adults’ understanding of mental health as a tool for schools’ health promoting work starting in the early school years, it would be interesting to conduct a similar study among children with younger ages. 

## 5. Conclusions

Research investigating young people’s perspectives regarding their own lived experiences is scarce, and listening to the voices of young adults could be a way to identify important skills and abilities for adaptive and positive behaviour that will enable youth to deal effectively with the demands and challenges of everyday life. In this study, the most pronounced life challenges voiced by the young adults included academic failures, negative self-evaluations through social comparisons, relationship problems, and other performance-oriented tasks that result in stress, negative feelings, and, worst case, mental ill-health. The challenges to students’ well-being and mental health are many, and there are no simple solutions. 

Based on the results in this study, life skills training should include elements to enhance the development of individual coping strategies to apply when life feels tough and when the body is experiencing stress reactions. Most importantly, youth may need to be exposed to setbacks and experiences of failure, and not try to avoid them, in order to develop independent coping strategies. This also includes strategies for help-seeking behaviour. Further, to minimize the risk of self-stigma and the internalization of negative stereotypes and self-blame, life skills training should include elements to increase knowledge of structural factors that have effects on the life situation among youth. Young people’s reactions may simply be healthy reactions in an unhealthy environment. In addition, life skills training should also include parents, school personnel, and other important adults in the lives of the young people. Teachers, schools, and parents can make a real difference, and together they can attend to students’ psychological and social needs, helping them develop a sense of control over their future and the resilience they need to be successful in life [12]. 

## Figures and Tables

**Table 1:** Qualitative content analysis showing examples of adolescents’ and young adults’ views on mental health.

Meaning-Carry Unit	Condensed Meaning-Carry Unit	Code	Sub-Category	Category
“A classic example is this… well after the test… that many people like to walk around and ask "what score did you get?" and then you feel pressure if you did not reach the same level or so. And that in turn can lead you to feeling unsuccessful and then you skip the next test instead…”	You feel pressure and unsuccessful if you did not perform as well as your classmates	Constant comparisons	Sources of mental health problems	Life challenges
“That it’s supposed to be good all the time and look nice… and that you can end up feeling bad if you don’t live up to that. I think that social media contributes to mental ill-health”	Social media creates expectations that everything in life is supposed to be good and nice all the time	Constant comparisons	Sources of mental health problems	Life challenges
”Because I can see friends that I love, who I wish all the success in the world… and catch myself and almost… be mad over their success and that they are experiencing fun things… ‘okay, so everybody else is doing great, why is everybody leaving me?” like that. And then it becomes very selfish, which I am well aware of and then you become mad at yourself…	Wanting your friends to succeed but at the same time feeling jealous and end up feeling ashamed for being selfish	Constant comparisons	Sources of mental health problems	Life challenges

**Table 2:** Showing the results in terms of categories and subcategories.

**Sub-categories**	**Categories**	
	**Life challenges**	**The need for present adults**
	Sources of mental health problems	A need to be seen and heard
	Managing relationships	A need for increased availability
	An accepted way to express mental health problems	-

## Data Availability

The data presented in this study are available on request from the corresponding author. The data are not publicly available due to ethical reasons.
